# North Staffordshire Infirmary: The Way to Get Money

**Published:** 1917-10-20

**Authors:** T. Basil Rhodes

**Affiliations:** Secretary and House Governor.


*To the Editor of* THE HOSPITAL.

### North Staffordshire Infirmary: The Way to Get Money.

*To the Editor of* THE HOSPITAL.

SIR,—Please allow me to thank you for your kindly and encouraging paragraph in THE HOSPITAL dated October 13, on page 25, with reference to the latest endeavour to obtain new and increased subscriptions in support of the North Staffordshire Infirmary.

As you have been good enough to notice the matter at all, I think you will be interested to hear that the appeal at this time is for two purposes—namely, for £10,000 to wipe off the overdraft which it was calculated would exist at the end of this financial year on October 25, and for an additional annual income of £10,000 to avoid a similar overdraft occurring at the end of each year.

The appeal is being organised carefully and thoroughly on the principle mentioned at the end of your paragraph; that is to say, with a view to its quiet and steady growth rather than to its suddenly flaring up and burning out.

I think it right to point out, however, that the appeal to employers is being made chiefly through their Associations, and the suggestion placed before them has been that each Association should consider and decide for itself what is the most suitable scale upon which its members should be invited to subscribe to the hospital.

I am happy to say that the matter is being taken up with generosity and enthusiasm, and we are all very hopeful of obtaining splendid results.

In addition to the endeavour to obtain new and increased subscriptions from the employers, the employees throughout the district are also being invited to commence subscriptions on a definite weekly scale in cases where this method of subscribing has not been in vogue, and to increase the scale to 1½d. per week for men and 1d. per week for boys and females, instead of the former scale of 1d. and ½d. per week respectively, in cases where a regular establishment subscription has been usually sent.

Kindly understand that I am not sending this letter necessarily for publication, but rather as an expression of thanks to you for the kindly notice you have taken of the effort in this district.—Yours faithfully,

T. Basil Rhodes, M.B., B.S.,

October 15, 1917. Secretary and House Governor.

[We are indebted to Mr. T. Basil Rhodes for this letter, which cannot fail to have a practical value for numerous readers of The Hospital engaged in administration and having special responsibilities in connection with hospital finance. The history and development of the North Staffordshire Infirmary, Hartshill, since October 1909, when we published an elaborate report and criticism on the state of affairs at this institution, as they then were, and pointed the way to reform, have proved as remarkable as they have proved successful and inspiriting. The efforts of the leading governors and those members of the staff who actively interested themselves in securing these results, including ample funds, new buildings, and the reform of the administration, culminated in the appointment of Mr. T. Basil Rhodes as secretary and house governor. Mr. Rhodes has proved a most active, energetic, able, and successful administrator. He has been wisely backed by the existing governors of the North Staffordshire Infirmary, and the results are amongst the most cheering and educative instances of how to raise a voluntary hospital to a high state of efficiency, to meet the ever-growing demand for increased financial support, and to maintain that efficiency in every department of its work. It is therefore not only a pleasure but a privilege to publish the encouraging letter, full of practical good sense and information, which precedes these comments.—Ed. The Hospital.]

